# Fabrication of Alginate-Based O/W Nanoemulsions for Transdermal Drug Delivery of Lidocaine: Influence of the Oil Phase and Surfactant

**DOI:** 10.3390/molecules26092556

**Published:** 2021-04-27

**Authors:** Omar Sarheed, Manar Dibi, Kanteti V. R. N. S. Ramesh, Markus Drechsler

**Affiliations:** 1 RAK College of Pharmaceutical Sciences, RAK Medical and Health Sciences University, Ras AlKhaimah 11172, United Arab Emirates; manar.dibi@hotmail.com (M.D.); venkatramesh@rakmhsu.ac.ae (K.V.R.N.S.R.); 2 Bavarian Polymer Institute, KeyLab ‘Electron & Optical Microscopy’, University of Bayreuth, 95447 Bayreuth, Germany; Markus.Drechsler@uni-bayreuth.de

**Keywords:** nanoemulsions, phase inversion temperature (PIT) method, oil type, surfactant concentration, in vitro skin permeation, pig skin, PDMS

## Abstract

Transdermal drug delivery of lidocaine is a good choice for local anesthetic delivery. Microemulsions have shown great effectiveness for the transdermal transport of lidocaine. Oil-in-water nanoemulsions are particularly suitable for encapsulation of lipophilic molecules because of their ability to form stable and transparent delivery systems with good skin permeation. However, fabrication of nanoemulsions containing lidocaine to provide an extended local anesthetic effect is challenging. Hence, the aim of this study was to address this issue by employing alginate-based o/w nanocarriers using nanoemulsion template that is prepared by combined approaches of ultrasound and phase inversion temperature (PIT). In this study, the influence of system composition such as oil type, oil and surfactant concentration on the particle size, in vitro release and skin permeation of lidocaine nanoemulsions was investigated. Structural characterization of lidocaine nanoemulsions as a function of water dilution was done using DSC. Nanoemulsions with small droplet diameters (d < 150 nm) were obtained as demonstrated by dynamic light scattering (DLS) and cryo-TEM. These nanoemulsions were also able to release 90% of their content within 24-h through PDMS and pig skin and able to the drug release over a 48-h. This extended-release profile is highly favorable in transdermal drug delivery and shows the great potential of this nanoemulsion as delivery system.

## 1. Introduction

Skin can act as a route of drug administration, but the stratum corneum, the outermost layer, is a major barrier for drug permeation [1,2]. The goal of transdermal drug delivery (TDD) is to achieve better percutaneous absorption by traversing the stratum corneum [3]. This mode of delivery is considered minimally invasive and painless, making it convenient for patient compliance [4]. Drugs can, however, be integrated into the TDD system if they meet the rule of five such as moderately lipophilic, low molecular weight and molecular size of less than 500 Daltons, optimal partition coefficient; preferably a logP value between 1 and 3 and a low melting point [5,6,7]. To overcome these challenges, various strategies, such as physical or chemical enhancement methods, are being investigated to increase skin permeation.

Physical enhancement involves the use of external energy to drive a drug molecule through the stratum corneum or to physically disrupt the barrier [3,8]. Chemical enhancement, on the other hand, includes the use of chemical penetration enhancers that would disrupt the highly ordered lipid intercellular matrix [3,9]. Nanocarriers, as a formulation strategy, have a great potential for molecule encapsulation and can be rendered soluble, such as microemulsions and nanoemulsions. Nanoemulsions are made up of two immiscible phases, one of which is dispersed in the other as droplets with a diameter of 100 to 500 nm. External energy is often needed for their preparation and the resulting formulation is usually transparent. They are also known to be metastable systems, since turbidity, creaming, or sedimentation are all signs of instability [10]. Nanoemulsions can be used to improve drug solubility and to protect them from hydrolysis and enzymatic degradation. [11].

Because of their smaller droplet diameter, nanoemulsions can withstand Brownian motion and gravity force as major sources of instability. Furthermore, the surfactants used in their formulation, allows homogenous distribution of droplets. They can also form a layer around the droplets to avoid coalescence and flocculation [12]. However, the main cause of instability in nanoemuslions is Ostwald repining, where large droplets grow at the expense of smaller droplets [13].

Anesthetics exert their action by targeting the sodium channels. In nerve cells, these channels play a role in nerve transmission, hence binding to the channel and blocking the permeation of sodium causes in reducing or eliminating sensation. Anesthetics consist mainly of a lipophilic aromatic ring and a hydrophilic tertiary amine bound by either a carboxyl ester or an amide. The tertiary form of the amine nitrogen makes it uncharged and thus more lipid soluble for better penetration [14]. Their absorption in the body usually depends on the site of application and its vasoactivity, rate of application and dosage. When distributed into the tissue, their distribution is proportional to the mass and perfusion of the tissue. Ester and amide anesthetics are metabolized by plasma hydrolysis and aromatic hydroxylation respectively by the liver. Thus, reduced hepatic flow can lead to the accumulation of the drug causing hepatic dysfunction [15].

Lidocaine, or lignocaine, is a synthetic aminoethylamide with local anesthetic and antiarrhythmic properties. The anesthetic property of lidocaine is exerted by its ability to bind to block sodium channels resulting in the inhibition of the depolarization phase of the action potential [16]. Lidocaine is usually administered by intravenous or hypodermic injection. Such applications cause discomfort so alternative routes such as transdermal delivery, have been explored. Lidocaine has a narrow therapeutic index and therefore extended release by TDD may also offer an increased safety profile [17]. Lidocaine, however, is found to display poor penetrative properties [18,19]. Several formulations have therefore been proposed to enhance its transdermal delivery such as liposomes [20] or microemulsions [21]. In those studies where microemulsions used, lidocaine concentrations ranged from 2–10% were investigated [22] with larger fluxes were achieved compared emulsion-based EMLA^®^ cream [23]. However, the use of higher concentrations of surfactants in microemulsions and alcohols as cosurfactants overweighs the advantages of higher lidocaine fluxes. More recently, nanoemulsions have been introduced as delivery system, to enhance lidocaine skin permeation and to minimize safety issues related to microemulsions as they require low concentrations of surfactants to form and without the need for cosurfactants to stabilize the system compared to microemulsions [21,22,23,24,25].

The aim of this work was to study the potential of lidocaine nanoemulsion as a transdermal drug delivery system and to evaluate its extended release profile. The alginate-based O/W nanoemulsion described by Sarheed et al. [26] has been employed as a transdermal delivery system for lidocaine and its release profile has been evaluated and compared to other formulations available in literature. Furthermore, the effect of formulation variables such as oil type, oil concentration and surfactant concentration, has also been studied. The lipids used included oleic acid, a cis-unsaturated free fatty acid [27]. Coconut oil was also used, which is composed medium-chain fatty acids, especially lauric acid, along with other fatty acids, such as oleic and linoleic acids [28]. Beeswax, on the other hand, is a natural fatty product obtained from the honeycombs of bees (*Apis mellifera*) [29]. Studies reported that oleic acid and lauric acid can cause disturbances in the startum corneum structure and thus enhancing transdermal penetration [27,30]. Additionally, there have been few reports on the use of beeswax in drug delivery systems. Furthermore, these oils are generally regarded as safe but are present in different physical states that can influence the behavior of the nanoemulsion. Tween 80 was chosen as the surfactant for the nanoemulsion because it is a non-ionic surfactant that will not irritate the skin when applied transdermally. In addition, it produces the lowest particle size compared with other Tweens [31]. These studies were carried out on both synthetic membrane and pig skin to gain better understanding of the behavior of the formulation as a transdermal delivery system.

## 2. Results and Discussion

### 2.1. Preparation and Particle Size Measurement

**Figure 1:**
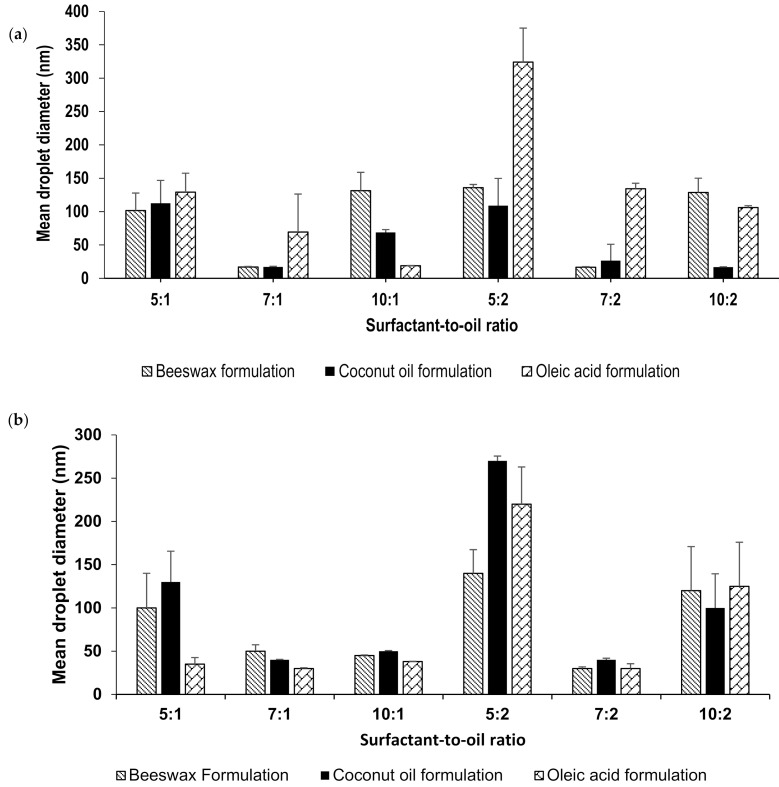
(**a**) Mean droplet size of fresh lidocaine nanoemulsions prepared at different SOR ratios and (**b**) Mean droplet size of lidocaine nanoemulsions after 7 months.

Lidocaine-loaded nanoemulsions were prepared by phase-inversion temperature method followed by ultrasonication with changing the surfactant-to-oil ratio (SOR); 5:1, 5:2, 7:1, 7:2, 10:1 and 10:2. Different lipids were added to the oil phase to produce different formulations which include beeswax, oleic acid and coconut oil. The combination of low-energy and a high-energy methods resulted in a stable transparent nanoemulsion. The formulation retained its physical appearance for a period of 7 months. This appearance indicates a stable formulation with no signs of instability such as creaming, precipitation, or crystallization. This was observed in all of the formulations prepared, wherein the transparency usually indicates the formation of small droplet size [26]. This was further demonstrated by DLS droplet size measurements. The mean droplet size was determined for all formulation by placing a sample of each nanoemulsion in the Litesizer without any dilution as they were very diluted (97.5% wt) so the effects of multiple scattering could be avoided [26]. Droplet size measurements are shown in Figure 1a, in which most formulations have a mean droplet size below than 150 nm. The increase in the surfactant concentration reduces particle size due to the decrease in the interfacial tension between the two phases. However, further increase in its concentration can lead to the aggregation of the excess surfactant and thus an increase in droplet size [32,33]. Similarly, increasing the oil concentration increased the droplet size [34]. Oleic acid nanoemulsions showed higher particle size, relative to formulations prepared with other lipids. The angular structure of oleic acid was proposed to cause larger particles [35]. Furthermore, owing to its long chain length, oleic acid non-polar property makes it less able to penetrate the interfacial film and less solubilized in the aqueous phase, resulting in larger particles [36,37]. Bees wax and coconut oil, on the other hand, contains polar components such as fatty alcohols and lauric acid, respectively [38,39]. This would lead to a decrease in the interfacial tension resulting in a smaller particle size [26]. Tween 80 was chosen as the surfactant for the nanoemuslion because it is a non-ionic surfactant that does not irritate the skin when applied transdermally.

The formulations were monitored periodically for any changes and was found to maintain their stability throughout the entire storage period at 25 °C; 6-months. Figure 1b shows droplet size measurements following 6-months in which most formulations still maintained a droplet size less than 150 nm, with the exception of those made at 5:2 surfactant-to-oil ratio.

The results from zeta potential measurements further prove the stability of the formulation. Results show high zeta potential value at an average of −71 mV over all formulations. According to literature, nanocarriers with zeta potential measured greater than ±30 mV are regarded as stable formulations, as evidenced by the transparent appearance of the nanoemulsion.

### 2.2. Drug Content and Encapsulation Effeciency

The actual amount of lidocaine in the nanoemulsion formulation was measured using HPLC and compared to the theoretical values. Lidocaine solubility in lipids was examined and was found to be the highest in oleic acid (406 mg/mL) followed by beeswax (347 mg/mL). Coconut oil showed the lowest solubility to lidocaine of 64 mg/mL [26].

The concentrations obtained are found to be similar to those calculated during preparation. Formulations with a higher oil concentration; 0.3 g, displayed an average concentration of lidocaine as 2.44 ± 0.15 mg/mL. While formulations that have been prepared with a lower oil concentration; 0.15 g, displayed an average concentration of lidocaine as 1.24 ± 0.06 mg/mL. Drug content results of all nanoemulsions are shown in Table S1.

On placing the nanoemulsion in the dialysis membrane and measuring the concentration of lidocaine released into the receptor compartment, it was found that the nanoemulsion had a high encapsulation efficiency for lidocaine. Entrapment efficiency was found to be comparable for all formulations and calculated at an average of 97% with no significant difference in encapsulation when the SOR was changed. This can be attributed to the hydrogen bonds formed between lidocaine and nanoemulsion ingredients including Tween 80 and alginate. These bonds are formed between the amine group of lidocaine and the hydroxyl groups of Tween 80 and alginate, improving encapsulation efficiency of nanoemulsions [26]. This might affect the release properties of lidocaine from nanoemulsions.

### 2.3. Cryogenic Transmission Electron Microscopy

**Figure 2:**
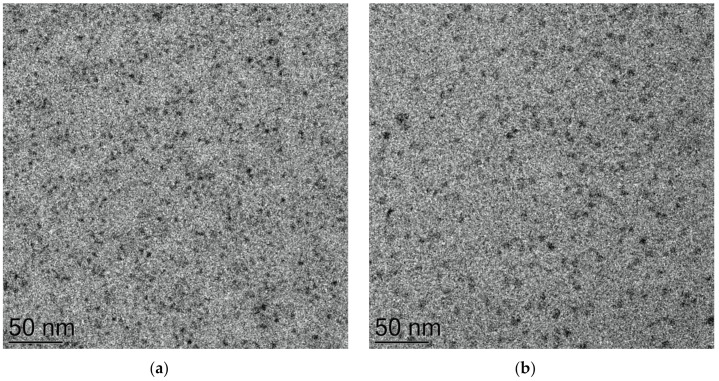
Cryo-TEM micrograph of (**a**) primary emulsion concentrate and (**b**) final nanoemulsion of coconut oil (SOR 7:2).

The nanoemulsions appeared transparent upon physical examination. Transparency often indicates a small droplet size, as demonstrated earlier by droplet size measurements. Droplets were further investigated by cryo-TEM and the findings were compared. Figure 2 shows the resulting micrograph of a representative nanoemulsion for a primary nanoemulsion; before dilution (Figure 2a) and of the final nanoemulsion (Figure 2b). A representative lidocaine nanoemulsion at a surfactant-to-oil ratio of 7:2 and coconut oil as the lipid was chosen. The sample appears homogenous with visible features of small vesicles in the size range of 50 nm, which is consistent with DLS measurement of the same representative coconut oil nanoemulsion of SOR 7:2 as shown in Figure 1a and confirming the formation of a nanoemulsion. Similar data have been reported earlier [40,41,42]. It is important to note that, the distribution of particle sizes obtained by cryo-TEM imaging might not be perfectly representing the whole sample with bearing in mind the technical differences between DLS and cryo-TEM in measuring the particle size [42]. Cryo-TEM reflects the actual sizes of the individual particles, whereas DLS gives the intensity-averaged values of hydrodynamic diameter, which can be skewed toward larger dimensions due to the higher scattering intensity of larger particles [42]. Cryo-TEM findings suggest the presence of Regime II due to the presence of nanosized droplets as well as swollen micelles as described in earlier reports [41,43]. It is worth noting that the primary emulsion concentrate tends to be more packed with smaller droplets than the final emulsion which displayed less density and a lower number of droplets as a result of the dilution process during the O/W nanoemulsion preparation (Figure 2a). Similar observations were reported by other groups [44,45,46].

### 2.4. Differential Scanning Calorimetry of Nanoemulsions

**Figure 3:**
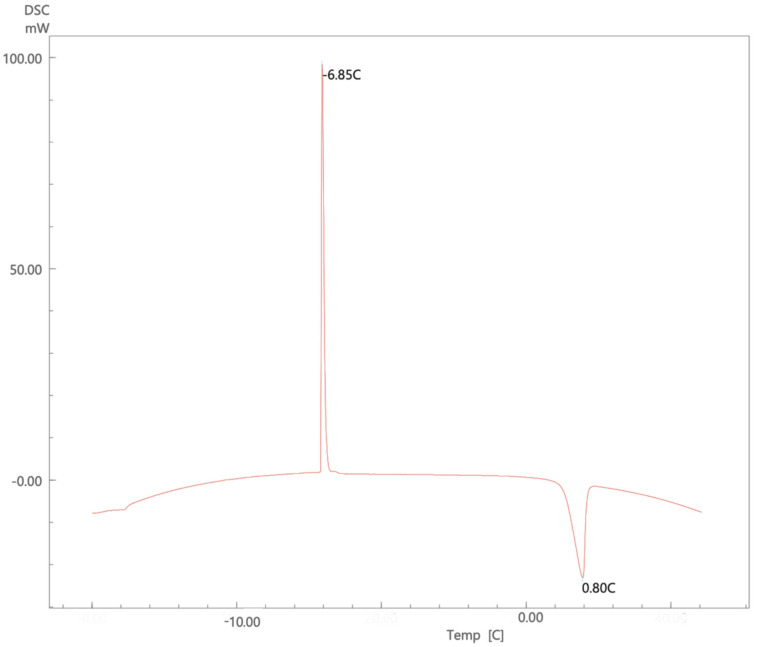
DSC thermogram of a representative lidocaine nanoemulsion using coconut oil at surfactant-to-oil ratio 7:2.

Studying the thermal behavior of the nanoemulsion is helpful to investigate the interactions between the water molecules and other components. Figure 3 presents a thermogram of a nanoemulsion that underwent a cycle of freezing followed by a reheating cycle. Similar to results reported by Dalmazzone’s group using DSC to characterize emulsified system, an initial exothermic freezing peak is noticed, which could be attributed to the crystallization that occurs upon freezing. This event happened fast and caused a significant amount of energy to be released in a short time causing the formation of the sharp freezing peak, then on reheating, the melting peak is observed at 0 °C. Dalmazzone et al. also proposes that the thermogram helps in determining the nature of the emulsion. In this case, the sharp freezing peak indicates an oil-in-water emulsion [47]. Additionally, the presence of sharp exothermic peak indicates that the system is monodisperse and confirms the cryo-TEM observation of system homogeneity [47]. The figure shows the result of a representative formulation that used coconut oil at a surfactant-to-oil ratio of 7:2. Similar thermograms were obtained for the other nanoemulsions (Figures S1–S9).

Figures S1 and S2 showed the thermograms of pure lidocaine, beeswax, oleic acid and coconut oil and their mixtures. There was a slight depression in the melting point of lidocaine present in the mixtures by 1–2 °C from its original one of 68.5 °C. When the oil phase was mixed with the water phase, the melting peak the oil phase of nanoemulsions was observed at a temperature about 23–31 °C lower than the lipid and drug mixture as shown in Figures S3–S8 suggesting that lidocaine located in the core of the nanoemulsion and had been successfully solidified by the phase inversion method (PIT) used. As previously reported by others [48], these results confirmed that nanoemulsions were prepared.

**Figure 4:**
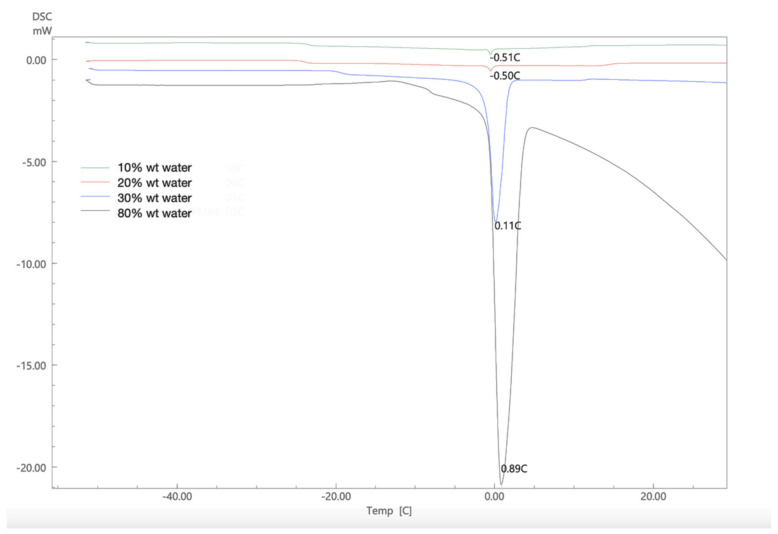
DSC thermogram of lidocaine nanoemulsion with increasing water content using beeswax at surfactant−to−oil ratio 7:2.

To understand the interactions that occur in the nanoemulsion between the water molecules and other components, the DSC thermogram is plotted for a representative nanoemulsion while adding water gradually. As shown in Figure 4, when the water content was below 30% wt the endothermic peak for melting transition of the water is barely detectable (−0.5 °C, ΔH = −3.16 J/g). It suggests that water molecules are bound with the Tween 80 and sodium alginate in the formulation. The presence of small amount of water in the surfactant mixture causes the formation of weak complexes, which makes the major endothermic events to appear at lower temperatures [49]. However, upon further addition of water a higher enthalpy of melting is measured along with the increase in the melting point, at 30% wt water (0.11 °C, ΔH = −147.95). This shift in the melting behavior is due to the dilution whereby water molecules become less bound with the surfactant but rather distributed throughout the formulation making the continuous phase enriched with water, at 80% wt water (0.89 °C, ΔH = −300.01 J/g). The enthalpy measured indicates the presence of bulk water which produces a large endothermic peak with prominent tail which was close to the measured enthalpy of water −216.98 J/g [45,50].

### 2.5. Permeation Studies

#### 2.5.1. In Vitro Release and Permeation Studies from Polydimethylsiloxane (PDMS) Membrane

Lidocaine nanoemulsions showed an initial burst release of lidocaine which can be attributed to drug movement from the drug-enriched core (Figures S9–S11) and the core formation can be explained by the mechanism of lipid precipitation during cooling. It is proposed that drug supersaturation occurs during the process of cooling of the nanoemulsion template, which induces precipitation of the drug before that of the lipid. This allows the precipitating lipid to encapsulate the precipitated drug, resulting in the formation of a membrane surrounding the drug [51]. Statistically, there were no significant effects (*p* > 0.05) on drug release because of oil phase composition and surfactant concentration except when the oil phase composition is coconut oil which can be attributed to the solubility of lidocaine and the structural integrity of the lipid which makes the drug take longer to diffuse out from the inner core to the outer medium [40]. The nanoemulsions produced in this study had a low viscosity and this expected to result in fast release of lidocaine. Similar results were reported by Yuan [22].

**Figure 5:**
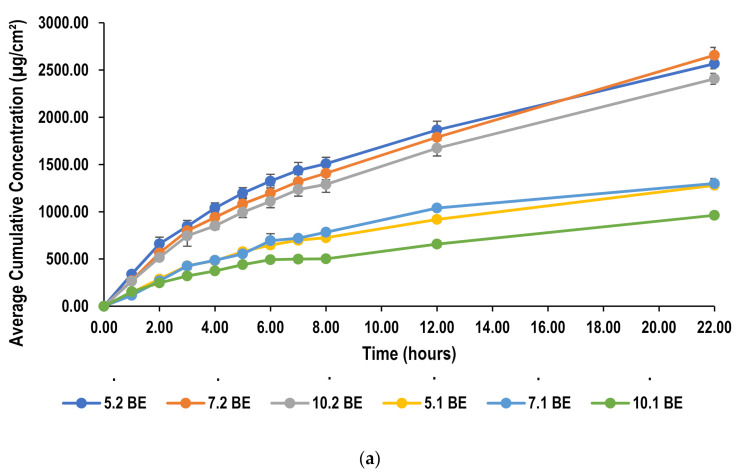

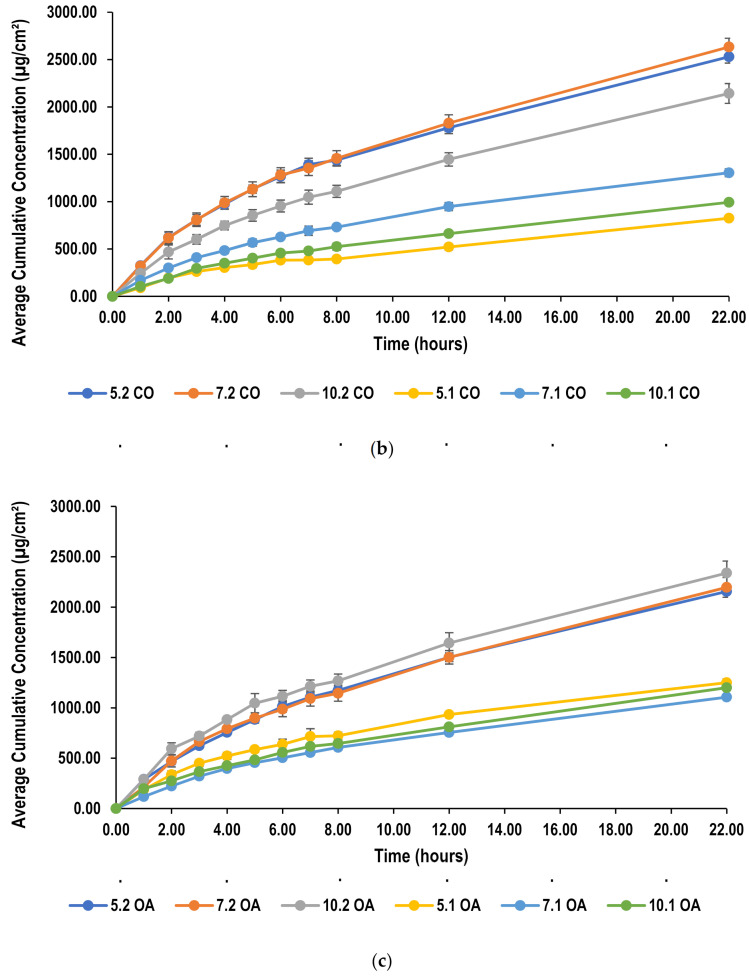
Permeation profile of lidocaine through PDMS from (**a**) beeswax formulations (**b**) coconut oil formulations and (**c**) oleic acid formulations.

The permeation of lidocaine from all nanoemulsions is presented in Figure 5, which shows the cumulative amount of lidocaine that permeates across PDMS membrane as a function of time over a period of 22 h. Although, PDMS membrane often provides good correlation with an in vivo situation, it is considered to offer a rather simplified skin model. This seems to be advantageous while demonstrating formulation screening and drug permeability, as this synthetic membrane produces reproducible results, reduces cost and eliminates any ethical issues as compared with natural skin [52]. Yet, there are certain disadvantages to the use of PDMS; for example multiple factors could affect the permeation such as ionization whereby permeation is more favorable for the more unionized form of a compound [53]. Receptor solution seems also to affect drug permeability as suggested by Shahzad et al. [54]. The selection of an appropriate receptor solution is usually based on the solubility of a permeant and its sink condition to ensure solubility does not become a rate limiting factor in permeation. However, it was proposed that an ethanol-water mixture in the receptor compartment seem to produce a higher permeation than any other receptor medium [54].

Nanoemulsions and microemulsions showed great potential for transdermal drug delivery. They provide a method to improve drug solubility for both lipophilic and hydrophilic molecules, thus, encapsulate high concentration of drug. Additionally, they are favorable for their ease of preparation. Moreover, it is proposed that their use for transdermal drug delivery enhances the permeability profile due to the smaller droplet size which increases the contact with the membrane improving the delivery of the encapsulated drug [55,56]. However, it has been reported that nanoemulsions achieve a higher degree permeation and drug loading capacity as compared with microemulsions [10,25,57]. Furthermore, an advantage nanoemulsions possess over microemulsions, is the use of less surfactant concentration whose increase may lead to skin irritation [40].

Permeation parameters including steady-state flux (*J*), and permeability coefficient were calculated from the data obtained from Franz diffusion cell studies and are summarized in Table 1. Ideally, the same molecule produces the same flux values across the membrane regardless of the composition of the formulation in the donor compartment. However, this holds true if the other formulation components did not interact with the membrane. As given in Table 1, the flux values of lidocaine vary in some cases with different surfactant concentration, oil concentrations and oil type. This change could be attributed to the interactions between either the surfactant and membrane or the drug and surfactant [52].

Both flux values and cumulative amounts permeated of liodcaine were calculated and compared with those found in literature to illustrate the ability of this nanoemulsion to achieve a more favorable permeation of lidocaine. Bhuiyan et al. showed highest flux of 64.84 µg/h/cm^2^ using non-ionic surfactants, namely Tween 80 and Brij 35, which was similar to this study at a lower surfactant concentration. At higher surfactant concentration, however, flux became higher which will be discussed later. On another note, the maximum amount that was able to permeate through the membrane after 6 h was found to be 394.04 µg/cm^2^, whereas in this study reached higher than 1000 µg/cm^2^ [52]. Waters et al. also showed similar results in which the highest flux value obtained was 118.4 µg/h/cm^2^ which is similar to those obtained in this study. The cumulative amount permeated, on the other hand, were still lower than those permeated using this formulation [53]. Similarly, in other published literature flux values were found to be comparable with that presented in literature while producing a higher cumulative concentration [58]. Additionally, the permeation achieved with this nanoemulsion was able to be maintained over a period of 22 h. This extended release profile might be attributed to the use of sodium alginate in the formulation [26].

#### 2.5.2. Effect of Oil Concentration on Permeation of Lidocaine through PDMS Membrane

It was found that an increase in oil concentration resulted in an increase in flux values. In this formulation, lidocaine was added to the lipid making it a part of the oil phase. Thus, an increase in the oil phase concentration resulted in an increase in both the lipids added to the formulation as well as the drug concentration. A higher lidocaine concentration in the donor compartment results in an increase in the concentration gradient across the membrane which could be the reason behind higher flux values [21].

#### 2.5.3. Effect of Surfactant Concentration on Permeation of Lidocaine through PDMS Membrane

**Figure 6:**
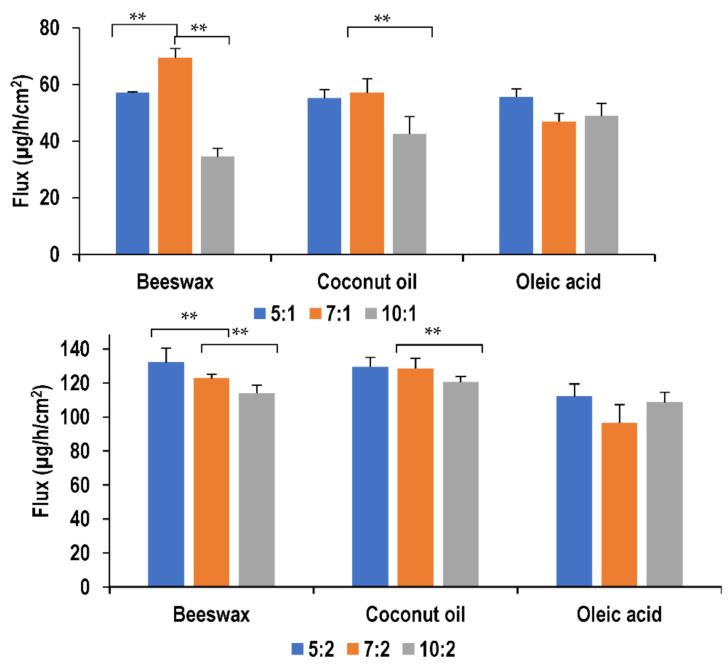
Effect of surfactant and oil concentration on flux of lidocaine nanoemulsion through PDMS membrane (** p > 0.05).

Nanoemulsions formulated using beeswax showed significant differences (*p <* 0.05) in flux with different surfactant concentration. This was observed only at a lower oil concentration, where the initial increase in surfactant concentration from surfactant-to-oil ratio of 5:1 to 7:1 resulted in an improvement in drug permeation. On the other hand, further increase in surfactant concentration had a retardation effect on the drug permeation. Similarly, using coconut oil as the lipid, increasing surfactant concentration also resulted in decreasing the flux. Coconut oil formulations of surfactant-to-oil ratio 5:1 and 7:1 showed similar flux values (*p >* 0.05) which where both higher than 10:1. This can be explained by the fact that an increase in surfactant concentration causes more lidocaine to be absorbed due to its surface-active effect and thus larger transdermal fluxes result. However, increasing concentration over a certain limit retards the permeation as reported by Yuan et al. [23]. A change in surfactant concentration did not seem to affect oleic acid formulations (*p >* 0.05) of both oil concentrations. This behavior is similar to all formulations at higher oil concentrations. Figure 6 illustrates the effect of these parameters on flux values.

#### 2.5.4. Effect of Oil Type on Permeation of Lidocaine through PDMS Membrane

**Figure 7:**
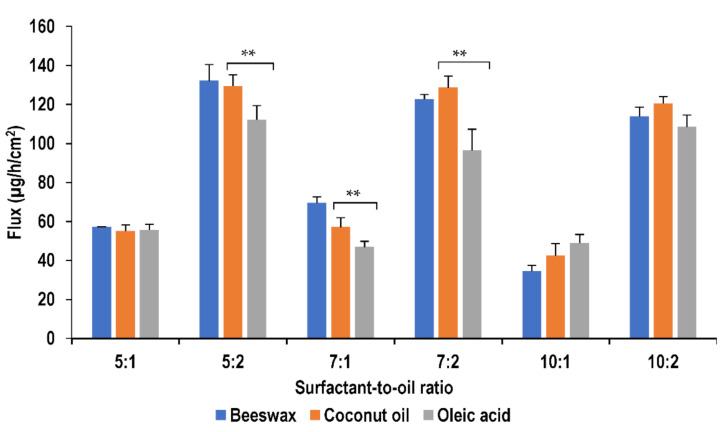
Effect of oil type on flux of lidocaine nanoemulsion through PDMS membrane (** *p >* 0.05)

The change in type of lipid seems to influence the permeation of lidocaine as shown in Figure 7. At surfactant-to-oil ratios of 7:1, 7:2 and 5:2, oleic acid formulations showed the lowest permeability compared with other lipids (*p <* 0.05), while coconut oil and beeswax formulations showed no significant difference (*p >* 0.05) in permeation. This decrease in permeation with the presence of oleic acid could be explained by the solubility of lidocaine in oleic acid which was found to be the highest relatively [26]. It is proposed that higher drug solubility typically results in lower drug permeability [21,59]. However, at higher surfactant concentrations at ratios of 10:1 and 10:2, no significant difference was found in the permeation of the nanoemulsions with change in lipid type. The higher concentration of surfactant seems to negate the influence of oil on drug permeation.

#### 2.5.5. Skin Permeation Studies

**Figure 8:**
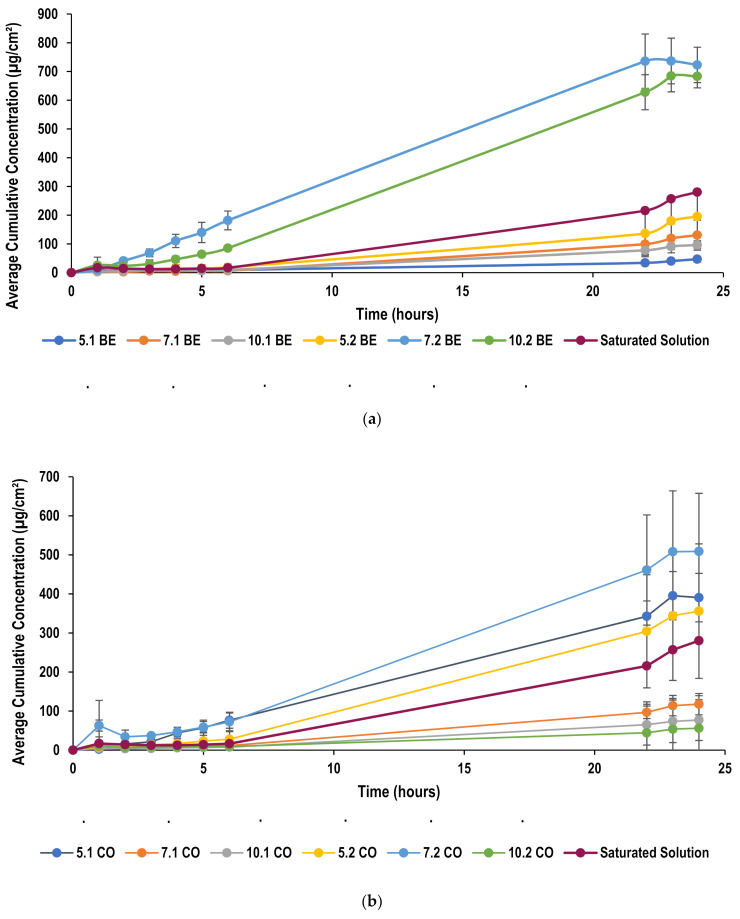

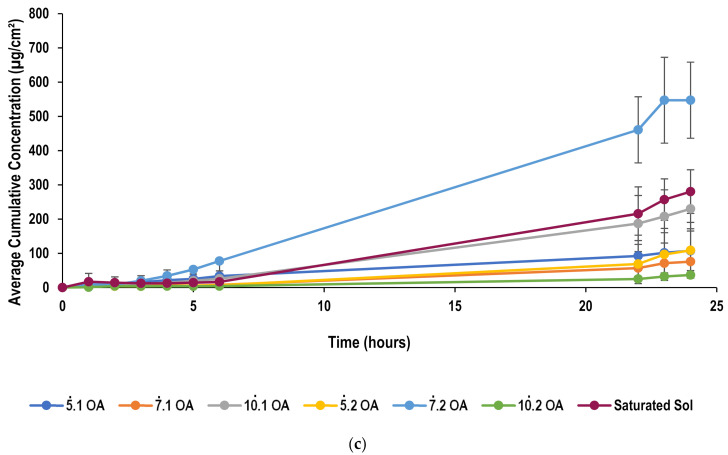
Permeation profile of lidocaine through pig ear skin from (**a**) beeswax formulations (**b**) coconut oil formulations and (**c**) oleic acid formulations.

The transdermal permeation profile of lidocaine from nanoemulsions is given in Figure 8, which shows the cumulative amount of lidocaine that permeates across pig ear skin as a function of time over a period of 24 h. Pig skin has been selected due to the ethical considerations that surround the use of human skin [60]. Furthermore, it has been found that certain variations occur among human skin specimens. Such variations are found to be due to differences in age, race, and anatomical donor site [61]. Alternatively, animal skin can be used to perform the permeation studies which will pose less ethical issues, is easier to obtain and exhibits less variability due to the use of inbred animal strains. However, the selection of animals’ skin should be that it is a good representative of human skin. Rodent skin is found to be commonly used model as they are readily available and easy to handle [60]. Nevertheless, it has been reported that rat skin is more permeable than human skin leading to overestimation [62]. On the other hand, pig skin has been long proved to be histologically similar to human skin, thus, abundantly used as an animal model to demonstrate skin permeation in vitro [63].

Steady-state flux (*J*), and permeability coefficient values are summarized in Table 2, which were calculated from the data obtained from Franz diffusion cell studies following analysis by HPLC.

**Figure 9:**
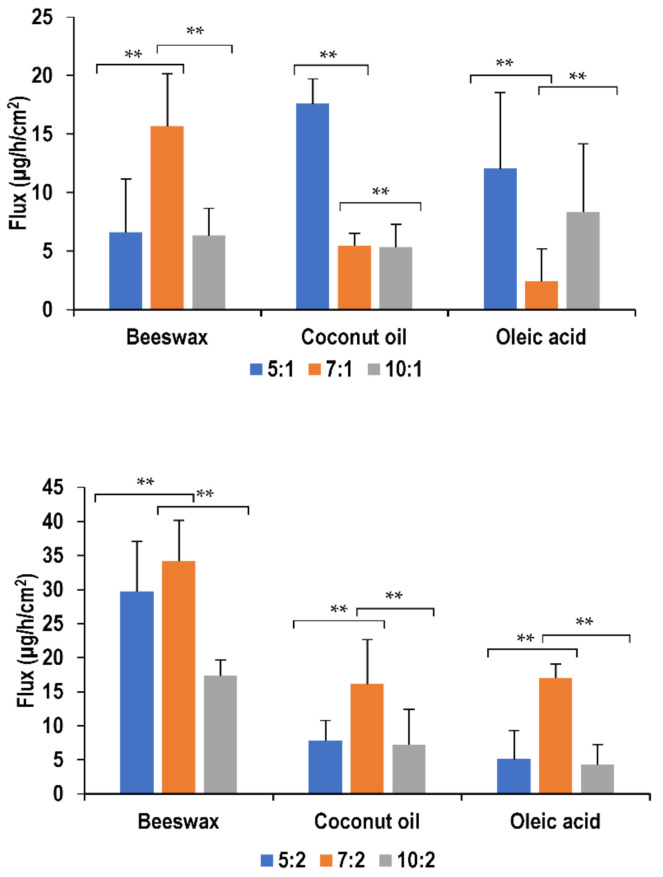
Effect of surfactant and oil concentration on flux of lidocaine nanoemulsion through pig skin membrane (** *p >* 0.05).

A saturated solution of lidocaine was prepared by dissolving excess amount of lidocaine in water and mixing it with magnetic stirrer overnight. The solution was then filtered and kept in Franz cell to assess permeation profile. It is noticed that the use of nanoemulsion formulation produced an enhanced permeation as compared with the saturated solution which could be attributed to the nano-sized droplets which would have an increased surface area that can increase the contact of the encapsulated drug with the stratum corneum producing improved permeability for transdermal delivery [55]. As discussed earlier, the change in flux could be as a result of the interactions between the surfactant and membrane or the drug and surfactant. The flux increases with the increase in oil concentration due to the higher concentration gradient that drives the lidocaine across the skin. Figure 9 demonstrates the influence of different surfactant and oil concentrations on flux values.

#### 2.5.6. Effect of Oil Concentration on Permeation through Pig Skin

The flux was found to increase with the increase in oil concentration due to the higher concentration gradient that drives the lidocaine across the skin. As discussed earlier, the increase in the oil phase concentration leads to an increase in amount of lidocaine added to the formulation as it is a part of the oil phase. Hence, the concentration gradient that drives the drug to permeate through the skin increases and flux increase as a result. However, some exceptions have been found, namely, nanoemulsions formulated at a surfactant-to-oil ratio of 5:2 using oleic acid and coconut oil when compared with those at a ratio of 5:1. Similarly, raising the ratio from 10:1 to 10:2 using oleic acid as lipid did not seem to increase the flux values (Figure 9).

#### 2.5.7. Effect of Surfactant Concentration on Permeation through Pig Skin

In beeswax formulations, permeation was found to improve significantly (*p <* 0.05) with initial increase in surfactant concentration from ratio 5:1 to 7:1, and then permeation has reduced with further increase in surfactant concentration, at ratio of 10:1. This behavior is similar to that noticed in PDMS permeation of the same formulations. Similarly, at a higher oil concentration, formulations of ratio 5:2 and 7:2 showed similar flux (*p >* 0.05) which was higher than that of formulations at a ratio of 10:2.

The same behavior has been observed in coconut oil formulations, whereby formulations at ratios of 7:1 and 10:1 showed reduced permeation as compared with 5:1 nanoemulsion. Also, permeation reduced significantly after changing the ratio from 7:2 to 10:2.

In oleic acid formulations, the highest flux was found at a ratio of 5:1 when compared with formulations of ratios of 7:1 and 10:1. At the higher oil concentration, again flux improves with initial increase in surfactant concentration and then reduces with further increase in surfactant concentration. These results further prove that the increase of surfactant concentration over a specific limit retards the permeation of lidocaine as presented in literature [52,60]. These observations confirmed the benefit of PDMS membrane in screening the formulation variables before testing on the skin.

#### 2.5.8. Effect of Oil Type on Permeation through Pig Skin

**Figure 10:**
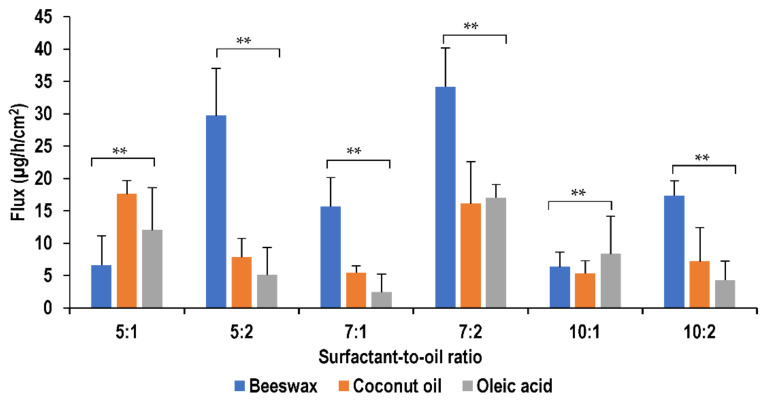
Effect of oil type on flux of lidocaine nanoemulsion through pig skin membrane (** *p >* 0.05).

The flux values of the nanoemulsions were found to vary with the change in the lipid type of the oil phase. It was found to be the highest using beeswax in most nanoemulsions including those formulated at surfactant-to-oil ratios of 7:1, 5:2, 7:2 and 10:2. Whereas, it was found to be the least in most of the oleic acid formulations, namely at surfactant-to-oil ratios of 5:1, 5:2, 7:1 and 10:2. Nanoemulsions prepared using coconut oil were found to show flux values mostly intermediate between those of oleic acid and beeswax as shown in Figure 10.

Beeswax contains a mixture of fatty alcohols and is expected to have higher solubility parameters for lidocaine and would thus be the most effective enhancer, as suggested by Kim et al. [64]. For oleic acid, the permeation enhancement is expected to result from the high solubility of lidocaine in oleic acid in the donor phase [64]. However, the lidocaine flux was smaller than of that of beeswax. This has been attributed to the presence of a high number of double bonds in oleic acid which cause steric hindrance, limiting the partitioning of the fatty acid to the lipid bilayers of the stratum corneum [64]. Coconut oil showed an intermediate effect on the flux. This may be due to the structure of coconut oil, consisting of higher levels of short chain saturated fatty acids such as lauric acid (which makes up about 50% of the fatty acids in coconut oil). These fatty acids have insufficient lipophilic characteristics to actively permeate the stratum corneum, thus, rendering poor penetration [40].

For all lipids, nanoemulsions formulated at a surfactant-to-oil ratio of 7:2 showed the most optimum flux values which was higher than that found in literature [21,22,23]. This was achieved at a lower lidocaine concentration in the nanoemulsion which is 0.24% as compared to that used in literature which is 10%. Wang et al. showed a higher flux value by approximately one-fold because the concentration of lidocaine used was 10% and permeation profile was studied on mice skin which causes an overestimation in permeability [55]. Nanoemulsions used in this study showed a potential by providing higher flux of lidocaine at lower lidocaine concentrations.

**Figure 11:**
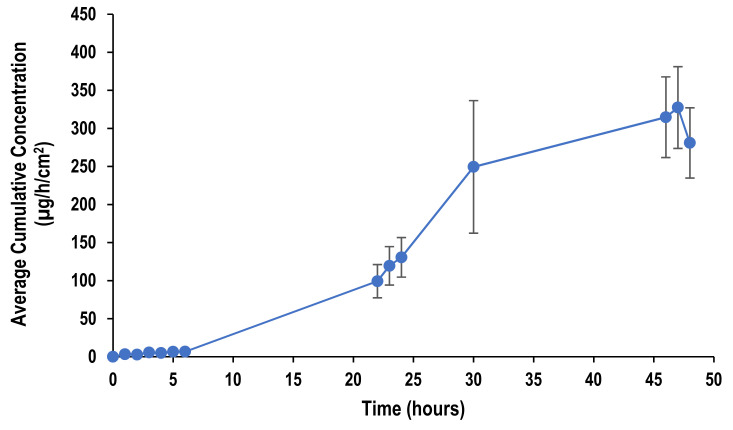
Permeation profile of lidocaine nanoemulsion through pig ear skin over 48 h.

Permeation of lidocaine from nanoemulsion was also studied for more than 24 h from a representative formulation to assess the extended-release ability of the formulation. The nanoemulsion used was formulated at surfactant-to-oil ratio 7:1 using beeswax as lipid. Figure 11 shows that lidocaine has maintained permeation for 47 h. The extended release achieved by the nanoemulsion is attributed to the addition of sodium alginate into the formulation.

### 2.6. Adsorption Studies

**Figure 12:**
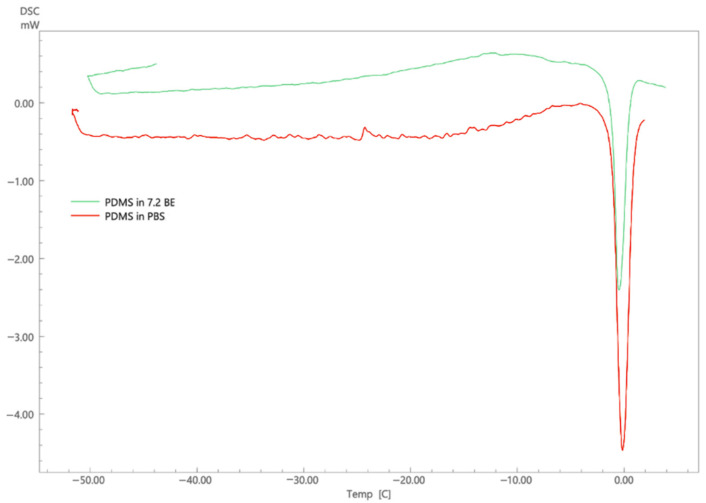
DSC thermogram of PDMS membrane.

Following soaking of PDMS membrane in lidocaine formulation, the amount of drug left was found to be similar to that of the formulation. On assessing the drug loss by UV spectrophotometric analysis, it was found that there was minimum loss in lidocaine due to interaction with the synthetic membrane, which accounts to less than 0.1 mg/mL. Also, DSC was used to assess any interactions between the PDMS membrane and the nanoemulsion. The thermogram of PDMS soaked in PBS pH 7.4 is compared with that of PDMS soaked in a representative nanoemulsion formulation. Figure 12 shows the thermogram of PDMS analysis. The peaks appeared at approximately same temperature and the enthalpy of PDMS soaked in buffer was found to be ΔH = −17.40 J/g, whereas the enthalpy of PDMS soaked in nanoemulsion was found to be ΔH = −13.69 J/g. Thus, no considerable difference was found in the DSC thermograms which indicates that there was no significant interactions between the nanoemulsion and the membrane, which was similar to findings of Bhuiyan [65]. Results from both experiments are hence found to be supporting each proving the absence of any significant interactions between the membrane and the formulation.

## 3. Materials and Methods

### 3.1. Materials

Lidocaine was donated by Gulf Pharmaceutical Industries (Julphar, Ras AlKahaimah, UAE). The lipids used were oleic acid, beeswax and coconut oil which were supplied by Avonchem, (Macclesfield, UK), Acros Organics, (Geel, Belgium), and LabChem Inc. (Zelienople, PA, USA), respectively. Tween 80 was purchased from Sigma-Aldrich, (St. Louis, MO, USA), and sodium alginate was obtained from Avonchem (Macclesfield, UK). The polydimethylsiloxane membrane (PDMS) utilized in the transdermal studies was purchased from Samco Silicone Products, (Nuneaton, UK). Chemicals for HPLC analysis included water for HPLC and were supplied by Fisher Scientific (Loughborough, UK) and acetonitrile and glacial acetic acid were purchased from VWR Chemicals BDH, (Lutterworth, UK).

### 3.2. Preparation of Nanoemulsions

The formulation of lidocaine nanoemulsion was previously proposed by Sarheed et al. [26], which was employed in this study. The components of the water phase; sodium alginate and Tween-80 were mixed and heated to 85 °C. The mixture was then added gradually to the previously heated mixture of drug and lipid with continuous homogenization using the Ultra-Turrax^®^ homogenizer (IKA T25, Staufen, Germany) at 8500 rpm for 5 min. 100 mL of previously heated distilled water to 85 °C was then added to the mixture while continuing the homogenization. Finally, the mixture was subjected to ultrasonic homogenization using a probe sonicator (300 V/T ultrasonic homogenizer, BioLogics Inc., Houston, TX, USA), at 20 kHz and power 70% for 5 min. The surfactant-to-oil ratio in the formulation was varied to determine its effect on the release behavior and skin permeation of the individual formulations. The amount of surfactant and oil added to the formulation and each corresponding surfactant-to-oil ratio is given in Table 3.

### 3.3. Particle Size Determination and Zeta Potential

A Litesizer 500 (Anton Paar Gmbh, Graz, Austria) was used to measure the droplet size of the nanoemulsions. The instrument uses the dynamic light scattering technique for measurement, which was set at back scattering angle of 175° and at 25 °C. The measurements were made in replicates of five, the mean and standard deviation of which were determined. Droplet size data was presented as the hydrodynamic diameter. The viscosity of 0.5% sodium alginate solution was 24 m Pa·s and was considered low to affect the DLS measurement. The samples were measured without dilution, as they were very diluted (97.5% wt.) so the effects of multiple scattering could be avoided [26].

Using the Litesizer, zeta potential was measured as well, which uses electrophoretic light scattering method. The sample was placed in an Omega cuvette, closed with the tips and placed in the measuring chamber. Measurements were made at temperature 25 °C. Each measurement was as a series of 3 repetitions per sample and the mean zeta potential and standard deviation were determined.

The effect of different surfactant-to-lipid ratios on the NEs stability was studied at room temperature (25 °C), over a period of six months. The dispersions were regularly examined for particle size as well as changes in physical appearance, such as gelation, precipitation, and crystallization.

### 3.4. Drug Content and Drug Entrapment Effeciency

To measure the amount of lidocaine in the formulated nanoemulsion, a sample of each formulation was analyzed by HPLC, method is discussed below. To characterize the entrapment efficiency of the nanoemulsion, dialysis membrane method was used. Cellulose membrane of 3500 Dalton molecular weight cut-off was used to enclose a nanoemulsion sample. This was tightly closed, kept in phosphate buffer pH 7 and allowed to stir overnight. The amount of lidocaine present in the phosphate buffer was measured by HPLC to identify the amount of unencapsulated drug thus, calculate the entrapment efficiency.

### 3.5. Cryogenic Transmission Electron Microscopy (Cryo-TEM)

For cryo-TEM studies, a sample droplet of 2 uL was put on a lacey carbon filmed copper grid (Science Services, Muenchen, Germany resp. Quantifoil R2/2, Quantifoil Micro Tools GmbH, Jena, Germany), which was hydrophilized by air plasma glow discharge unit (30 s with 50 W, Solarus 950, Gatan, Munich, Germany). Subsequently, most of the liquid was removed with blotting paper in a Leica EM GP (Wetzlar, Germany) grid plunge device, leaving a thin film stretched over the lace holes in the saturated water atmosphere of the environmental chamber. The specimens were instantly shock frozen by rapid immersion into liquid ethane cooled to approximately 97 K by liquid nitrogen in the temperature-controlled freezing unit of the Leica EM GP. The temperature was monitored and kept constant in the chamber during all the sample preparation steps. The specimen was inserted into a cryotransfer holder (CT3500, Gatan) and transferred to a Zeiss/LEO EM922 Omega EFTEM (Zeiss Microscopy GmbH, Jena, Germany). Examinations were carried out at temperatures around 95 K. The TEM was operated at an acceleration voltage of 200 kV. Zero-loss filtered images (ΔE = 0 eV) were taken under reduced dose conditions (100–1000 e/nm^2^). All images were registered digitally by a bottom mounted CCD camera system (Ultrascan 1000, Gatan) combined and processed with a digital imaging processing system (Digital Micrograph GMS 1.9, Gatan) [41,66].

### 3.6. Lidocaine Quantification

Samples taken from drug content permeation and entrapment studies and were analyzed by HPLC. An Onyx™ monolithic C_18_, 100 mm × 4.6 mm, 130 Å chromatographic column was used (Phenomenex Inc., Torrance, CA, USA). The column temperature was maintained at 25 °C and the injection volume was 20 μL. The pump used was a LC 20AD, (Shimadzu, Kyoto, Japan) and the detector was a SPD20A UV visible detector (Shimadzu, Kyoto, Japan).

The mobile phase consisted of two solutions eluted by gradient elution; the first was a mixture of H_2_O and glacial acetic acid of ratio 35:65 at pH 4.2 and the second was acetonitrile. 4 parts of the former was allowed to elute with 1 part of the latter through two pumps at a total flow rate of 0.5 mL/min.

To construct calibration curve, standard stock solution of lidocaine was prepared by weighing 25 mg of lidocaine, dissolved in 3 mL ethanol and then the volume completed to 25 mL with water to obtain a concentration of 1000 µg/mL. A series of dilutions were then prepared from the stock solution to obtain solutions of concentrations 0.05, 0.1, 1, 10, 50, 100, 200, 400 and 600 µg/mL and detected through its absorbance at 237 nm [67]. The calibration curve was plotted between AUC and concentration.

### 3.7. Differential Scanning Calorimetry (DSC)

Calorimetric analysis was performed using differential scanning calorimetry (DSC-60 plus, Shimadzu, Kyoto, Japan). A sample was taken from the bulk of the nanoemulsions ≈ 2–5 mg and placed in the aluminum pan which was sealed for analysis. The heat cycle applied was from −50 °C to 65 °C at a rate of 2 °C/min. DSC was also used to study the PDMS membrane assessing whether any of adsorption happened at the surface of the membrane. The same heat cycle described was implemented.

### 3.8. In Vitro Permeation Studies

#### 3.8.1. Polydimethylsiloxane (PDMS) Permeation Studies

The PDMS membrane is a synthetic membrane that was used to characterize the permeation property of lidocaine from nanoemulsion. The study was performed using Franz diffusion cells (V3A-02 PermeGear, Hellertown, PA, USA)

A mixture of phosphate buffer solution (PBS) pH 7.4 and ethanol absolute at a ratio of 80:20 (PBS:EtOH) was sonicated to remove air bubbles. The mixture was then added to the receptor compartment, which is 5 mL in volume. The mixture was continuously stirred with magnetic stirrer at 600 rpm and the systems temperature was maintained with thermostatic water pump (Haake SC 100, Thermo Fisher Scientific, Waltham, MA, USA) at 37 ± 0.5 °C. Temperature was maintained through circulating water through each chamber jacket.

The PDMS membrane was then mounted on the receptor compartment, which was previously soaked in PBS pH 7.4 and cut to fit the surface area of the compartment top. The membrane separates the donor compartment from the receptor compartment.

Over the PDMS membrane, the donor compartment was placed and tightly sealed with Parafilm M^®^. One mL of the formulation sample was placed in the donor compartment and sealed from top by the Parafilm M^®^ to avoid evaporation.

The study was run for 24 h, with a pre-determined sampling of 1 mL from the receptor compartment. The compartment medium was replenished with fresh medium of identical volume. The study for each formulation was done in triplicates to calculate the mean values and standard deviation.

The samples taken were analyzed in UV spectrophotometer (UV-1800, Shimadzu) at wavelength 262 nm and concentration was calculated using the calibration curve. Concentration values derived for each sample was corrected for the progressive dilution occurring during the course of the experiment. Cumulative amount of lidocaine over time was calculated and plotted against time. Flux values was calculated using the following equation. Permeability coefficient was noted as well:

(1) $$J_{ss}={\left(\frac{dQ}{dt}\right)}_{ss}\;.\;\frac{1}{A}$$

where *J_ss_* is the steady-state permeation flux (g/cm^2^/h), A is the area of skin tissue (cm^2^) through which drug permeates, and (*dQ*/*dt*)*_ss_* is the amount of drug passing through the skin per unit time at a steady state (g/h).

#### 3.8.2. Pig Skin Permeation Studies

Pig skin was allowed to thaw at room temperature before initiating the experiment. Parts of the skin was cut to fit the compartment top. The receptor compartment was filled with PBS:ethanol (80:20) and was maintained at a temperature of 37 ± 0.5 °C, and stirred with a magnetic stirrer at 600 rpm.

The skin was mounted on the compartment 30 min prior starting the study, to allow for skin hydration and system stabilization. The system was sealed with Parafilm M^®^ to minimize evaporation. Franz cells were inverted upside down to remove air bubbles that might formed during the hydration.

The donor compartment was placed on top of the skin and 1 mL of sample formulation was placed in the chamber. It was also sealed tightly with Parafilm M^®^. The study was continued for 24 h with hourly sampling of 1 mL from the receptor compartment and replenishing the chamber with fresh medium of identical volume. The study for each formulation was done in replicates of 6 to calculate the mean values and standard deviation. For extended release, the study lasted for 48 h. The release profile was calculated as a function of dosage (mg of nanoemulsion/cm^2^ skin).

The samples taken were analyzed by HPLC. Cumulative amount of lidocaine over time was calculated and plotted against time. The flux and permeability coefficient values were also calculated.

### 3.9. Adsorption Study

Certain amount of the drug may interact with PDMS membrane during the permeation study, in which the molecules may bind to the membrane leading to reduction in concentration. Thus, the adsorption of lidocaine onto the PDMS membrane is measured to assess for any loss in concentration during the permeation studies. PDMS membrane was soaked in PBS pH 7.4 and cut to the size of the surface area of the receptor compartment’s top. Fresh drug formulation was prepared and then the PDMS membrane is allowed to soak in 10 mL of the formulation for 6 h. The formulation was analyzed by UV spectrophotometer (UV-1800, Shimadzu) at a wavelength of 262 nm and the concentration of the formulation was calculated for both before and after the experiment. The difference in concentration is considered the amount of drug molecules bound to the membrane due to drug adsorption [68]. Additionally, the PDMS membrane both before and after soaking was kept for analysis by DSC and the resulting thermograms were compared with each other.

### 3.10. Statistical Analysis

Data obtained from the experiments was done in triplicates and the mean ± standard deviation was calculated. Statistical analysis was performed using Minitab software version 19 (Minitab, Ltd., Coventry, UK). Statistical significance was identified by performing student’s *t*-test where a *p* < 0.05 was considered to be statistically significant.

## 4. Conclusions

To conclude, the produced lidocaine-loaded nanoemulsion was proved by DSC to be an O/W nanoemulsion with the presence of bulk water in the continuous phase. The droplets formed in the emulsion with mean hydrodynamic diameter for most of the formulations was <150 nm. Cryro-TEM demonstrated the nanosized droplets of the emulsion. This nanoemulsion is intended to be used as a transdermal drug delivery system whose permeation profile through a synthetic membrane (PDMS) and pig skin was noted. Results showed a steady state flux comparable with those described previously in the literature, however, it allowed for the permeation of a higher cumulative amount of lidocaine for an extended period of time. The nanoemulsion demonstrated the ability to maintain the permeation of lidocaine through pig skin for a total period of 47 h. This extended-release profile is highly favorable for transdermal drug delivery and shows the great potential of this nanoemulsion as a delivery system. Characterization of the nanostructures will be further evaluated using cryo-TEM in future work.

## Figures and Tables

**Table 1:** Flux values and permeability coefficient for lidocaine nanoemulsions through PDMS membrane.

SOR	Lipid Type	Flux (*J*) µg/h/cm^2^	Permeability Coefficient cm/h
5:1	BE	57.08	0.57
	CO	55.14	0.27
	OA	55.70	0.56
7:1	BE	69.47	0.69
	CO	57.14	0.57
	OA	46.91	0.47
10:1	BE	34.51	0.35
	CO	42.53	0.38
	OA	48.93	0.49
5:2	BE	132.16	1.32
	CO	129.38	1.29
	OA	112.18	1.12
7:2	BE	122.66	1.23
	CO	128.55	1.29
	OA	96.56	0.97
10:2	BE	113.94	1.14
	CO	120.44	1.01
	OA	108.52	1.09

**Table 2:** Flux values and permeability coefficient for lidocaine nanoemulsions through pig ear skin.

SOR	Lipid Type	Flux (*J*) µg/h/cm^2^	Permeability Coefficient cm/h
5:1	BE	6.59	0.06
	CO	17.59	0.18
	OA	12.05	0.12
7:1	BE	15.67	0.16
	CO	5.45	0.05
	OA	2.42	0.02
10:1	BE	6.32	0.06
	CO	5.33	0.05
	OA	8.35	0.08
5:2	BE	29.71	0.30
	CO	7.83	0.08
	OA	5.09	0.05
7:2	BE	34.19	0.34
	CO	16.12	0.16
	OA	17.03	0.17
10:2	BE	17.34	0.17
	CO	7.20	0.07
	OA	4.30	0.04

**Table 3:** Concentrations of surfactant and lipids used in the preparation of lidocaine nanoemulsions.

Surfactant-to-Oil Ratio	Surfactant Amount (g)	Oil Phase Amount (g)
5:1	0.75	0.15
7:1	1.05	0.15
10:1	1.50	0.15
5:2	0.75	0.30
7:2	1.05	0.30
10:2	1.50	0.30

## Data Availability

The data presented in this study are available in Supplementary Material.

## Supplementary Materials

The following are available online. Figure S1: Differential scanning calorimetry curves of pure lipids, Figure S2: Differential scanning calorimetry curves of lidocaine and physical mixtures with lipids, Figure S3-S8: Differential scanning calorimetry curves of lidocaine nanoemulsion with different lipids, Figure S9–S11: Release study of lidocaine nanoemulsion formulated with (a) beeswax, (b) coconut oil and (c) oleic acid, Table S1: Drug content of lidocaine nanoemulsions.

## Sample Availability

Not available.
