# Supplementary material for: Fabrication of Alginate-Based O/W Nanoemulsions for Transdermal Drug Delivery of Lidocaine: Influence of the Oil Phase and Surfactant
Source: Molecules. 2021 Apr 27;26(9):2556. doi: 10.3390/molecules26092556 (PMC8125457; doi:10.3390/molecules26092556)
Supplement: Supplementary file 1 [file molecules-26-02556-s001.zip › molecules-1151186-supplementary.pdf]

Supplementary Materials:

Fabrication of alginate-based o/w nanoemulsions for transdermal drug delivery of lidocaine: Influence of the oil phase and surfactant

Omar Sarheed*, Manar Dibi, KVRNS Ramesh and Markus Drechsler

Table S1. Drug content of lidocaine nanoemulsions

Surfactant-to-oil ratio	Oil type	Drug content	Entrapment efficiency
5:1	BE	1.260 mg/ml	97.17 %
	CO	1.207 mg/ml	97.09%
	OA	1.241 mg/ml	97.10%
7:1	BE	1.300 mg/ml	97.00 %
	CO	1.268 mg/ml	96.00 %
	OA	1.207 mg/ml	97.00%
10:1	BE	1.160 mg/ml	97.10 %
	CO	1.391 mg/ml	97.35 %
	OA	1.109 mg/ml	96.00 %
5:2	BE	2.459 mg/ml	96.65%
	CO	2.668 mg/ml	97.12 %
	OA	2.193 mg/ml	96.72%
7:2	BE	2.486 mg/ml	96.95%
	CO	2.567 mg/ml	96.87%
	OA	2.289 mg/ml	97.10 %
10:2	BE	2.449 mg/ml	96.74%
	CO	2.540 mg/ml	96.86%
	OA	2.326 mg/ml	97.00 %

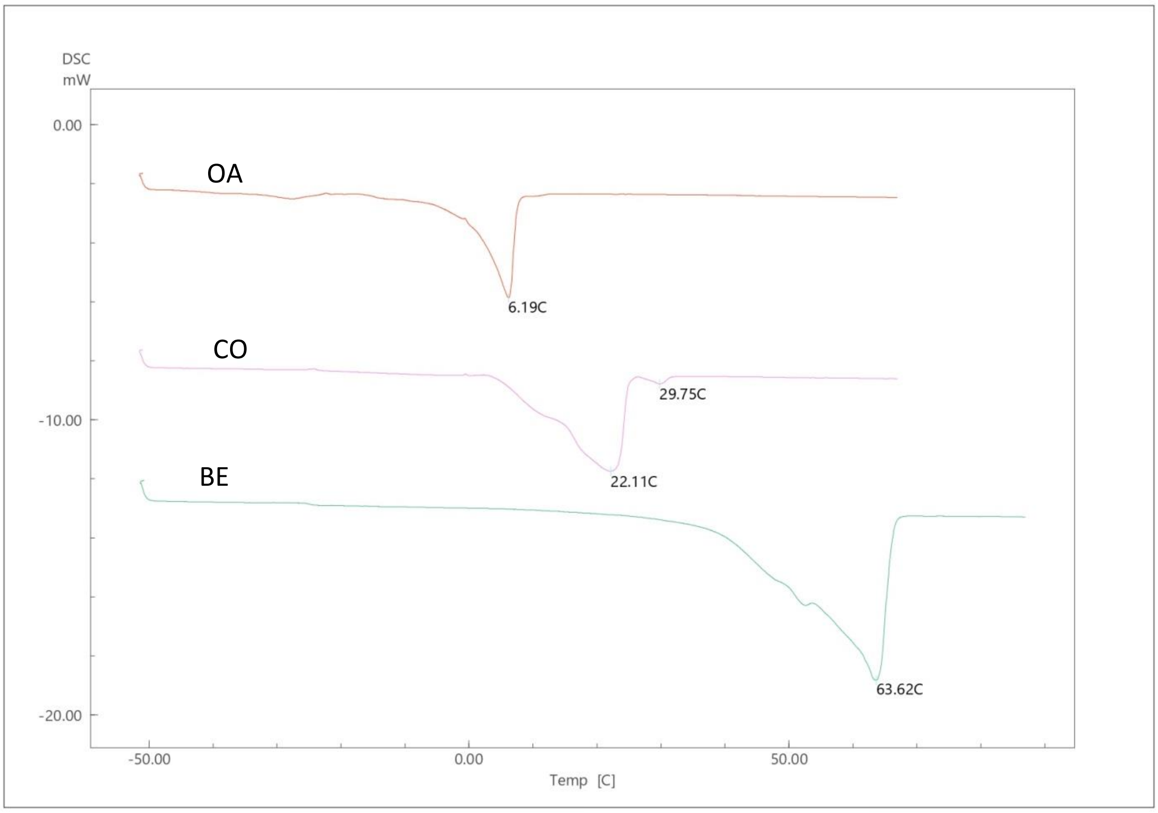

Figure S1. Differential scanning calorimetry curves of pure lipids.

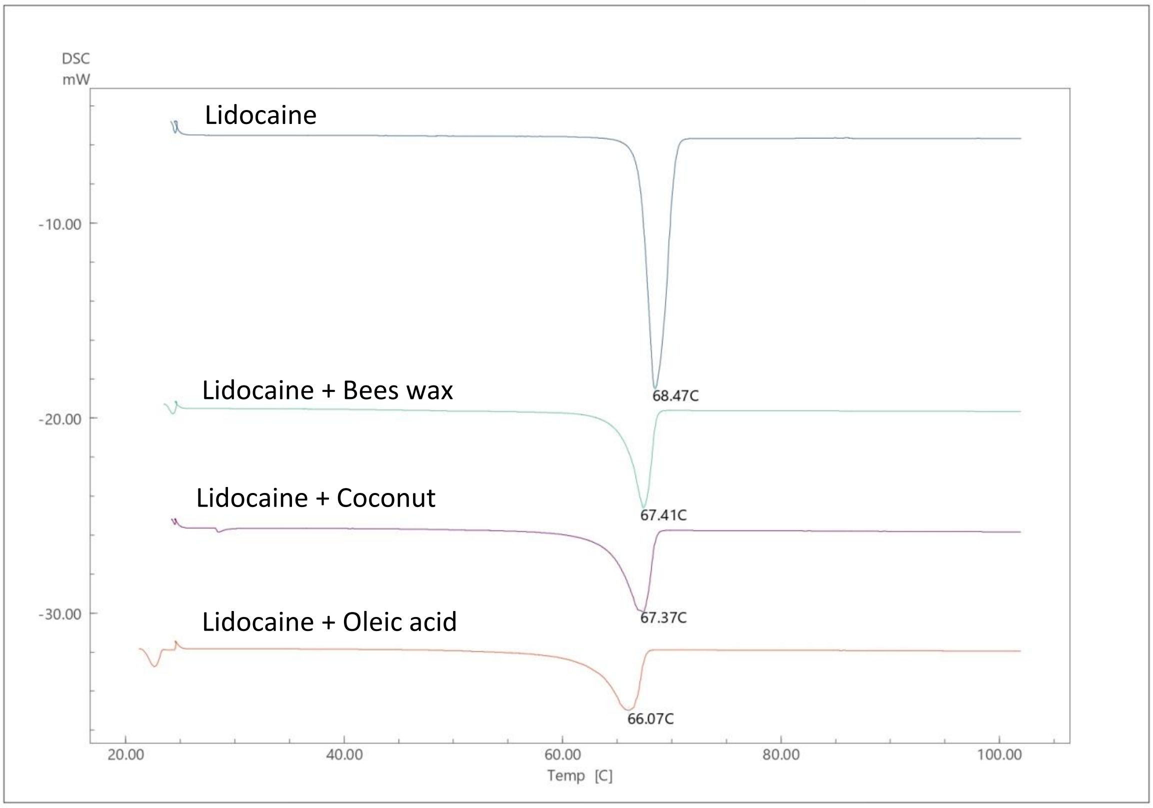

Figure S2. Differential scanning calorimetry curves of lidocaine and physical mixtures with lipids.

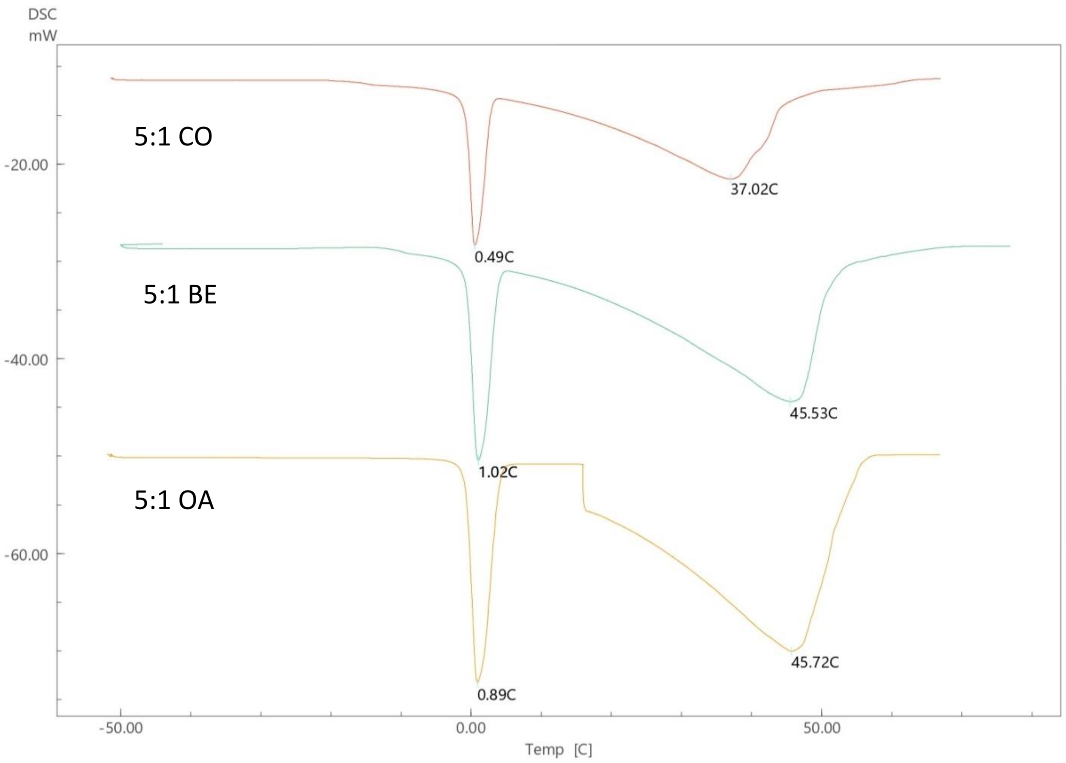
DSC
mW
5:1 CO
5:1 BE
5:1 OA
-20.00
-40.00
-60.00
37.02C
0.49C
45.53C
1.02C
45.72C
0.89C
-50.00
0.00
50.00
Temp [C]

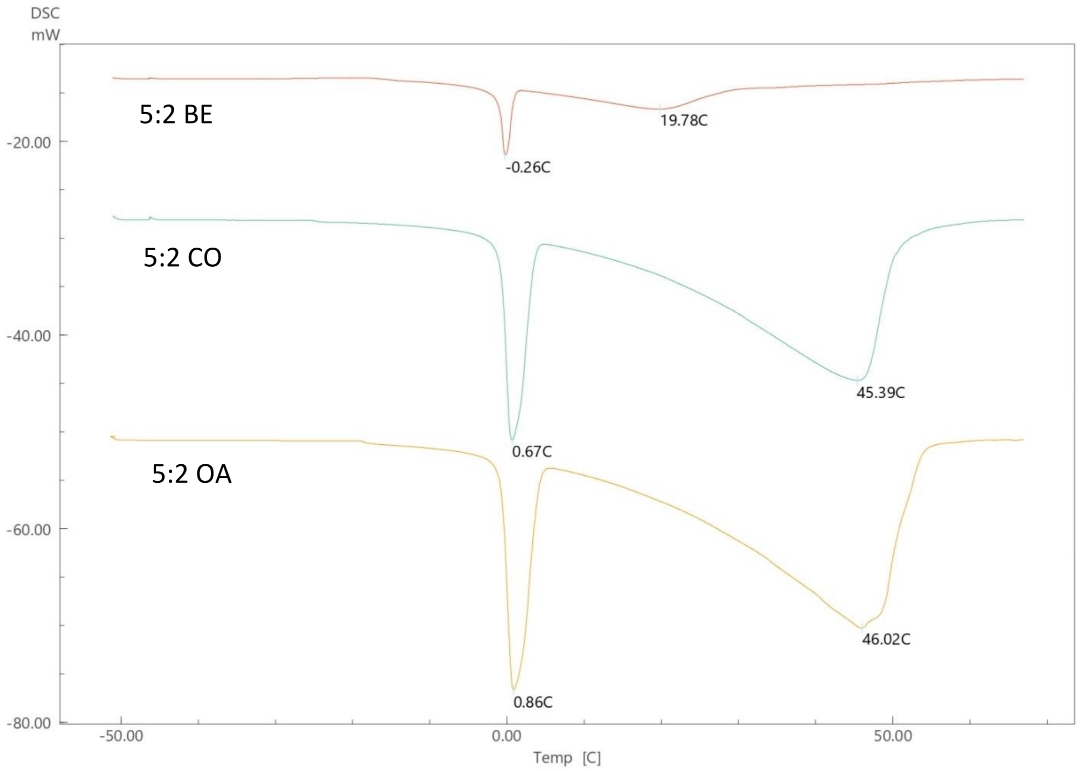
DSC
mW
5:2 BE
5:2 CO
5:2 OA
-20.00
-40.00
-60.00
-80.00
19.78C
-0.26C
45.39C
0.67C
46.02C
0.86C
-50.00
0.00
50.00
Temp [C]

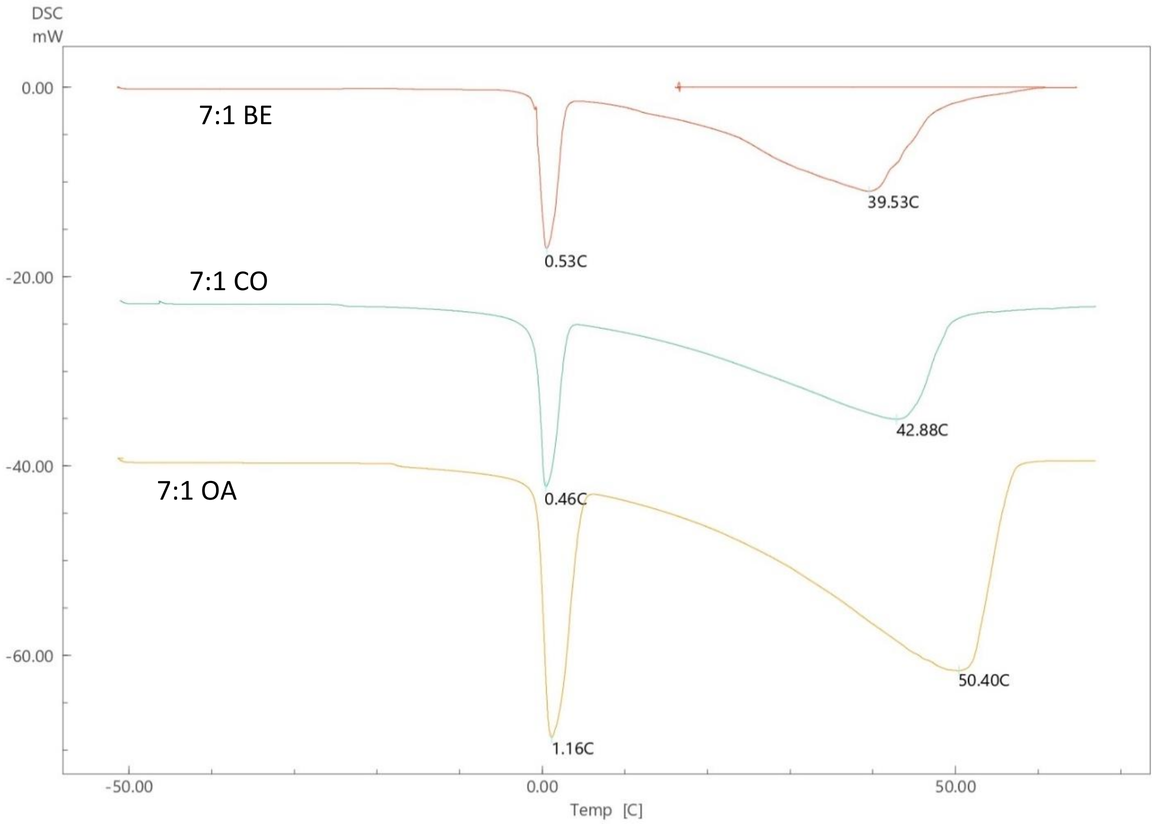
DSC
mW
0.00
-20.00
-40.00
-60.00
7:1 BE
7:1 CO
7:1 OA
0.53C
39.53C
42.88C
0.46C
50.40C
1.16C
-50.00
0.00
50.00
Temp [C]

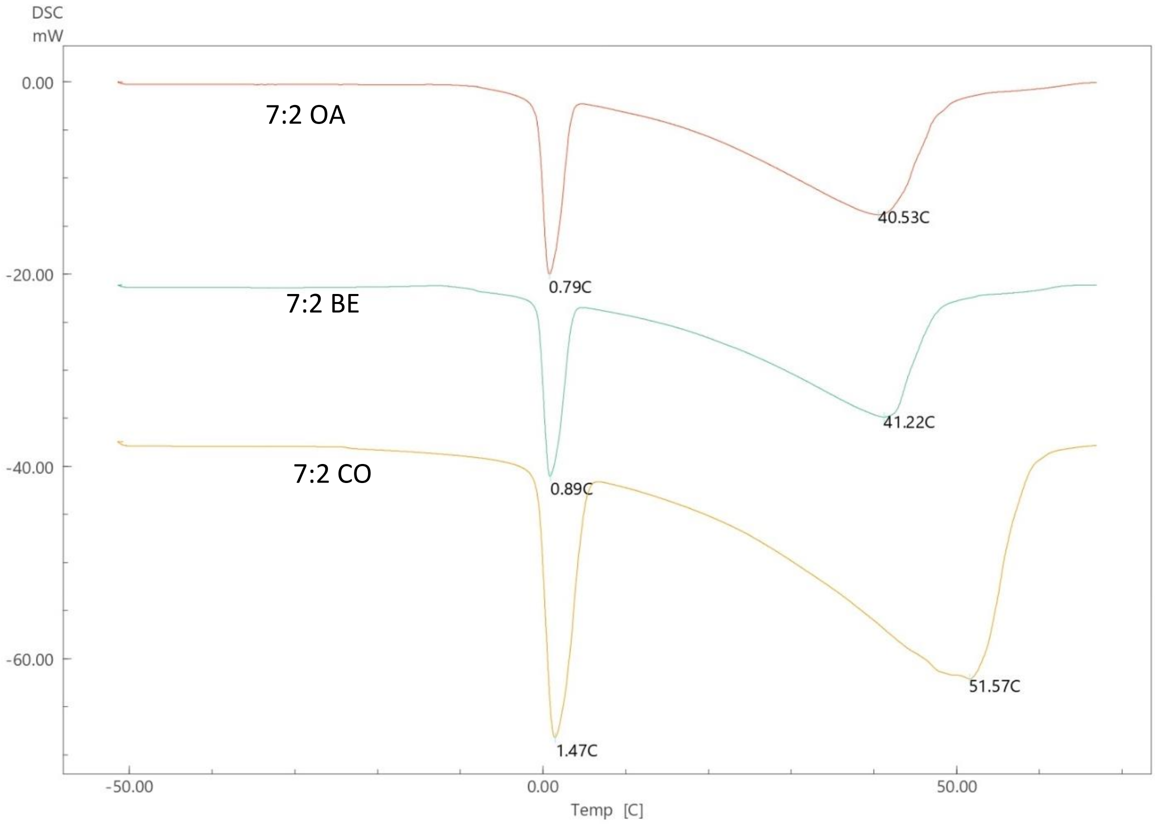
DSC
mW
0.00
-20.00
-40.00
-60.00
7:2 OA
7:2 BE
7:2 CO
40.53C
0.79C
41.22C
0.89C
51.57C
1.47C
-50.00
0.00
50.00
Temp [C]

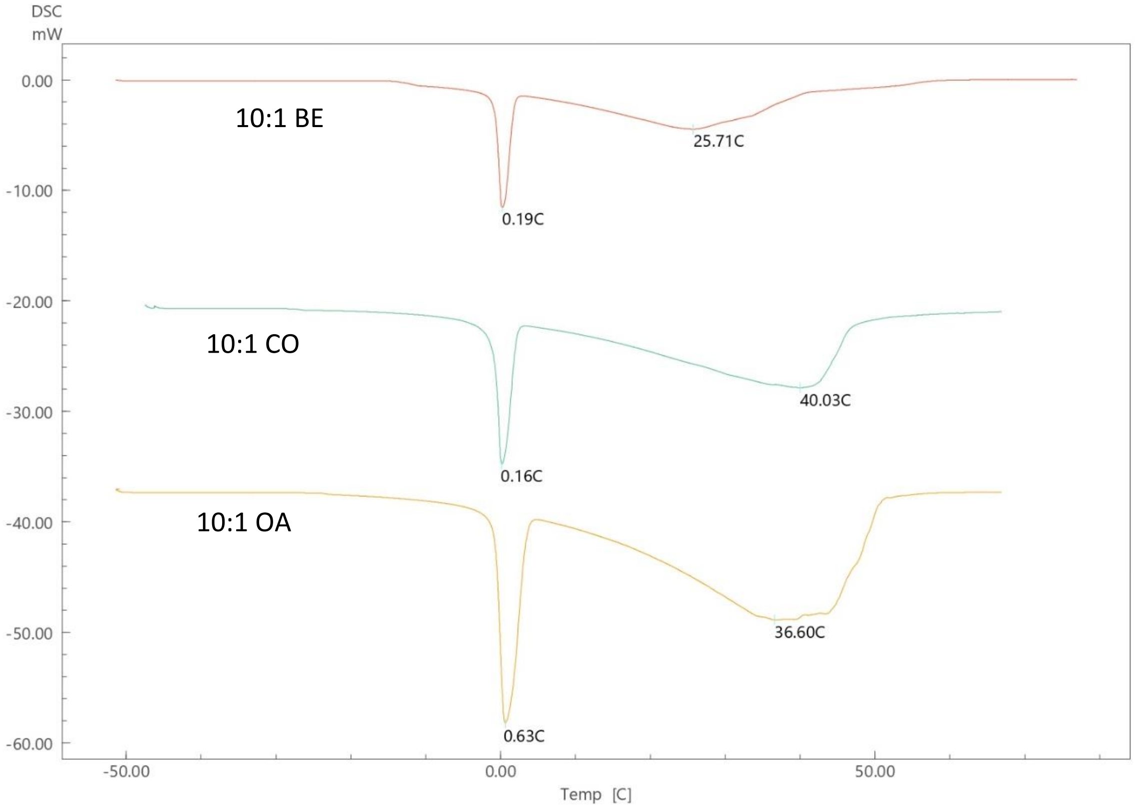

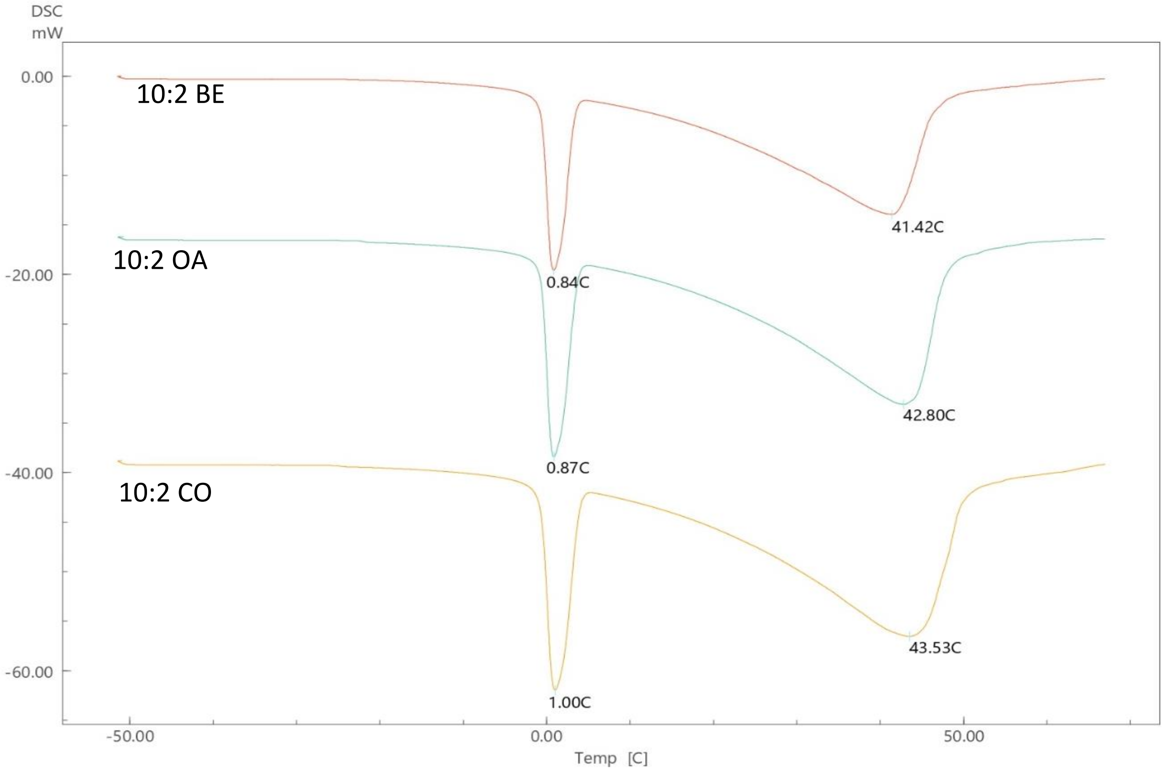

Figure S3-S8. Differential scanning calorimetry curves of lidocaine nanoemulsion with different lipids.

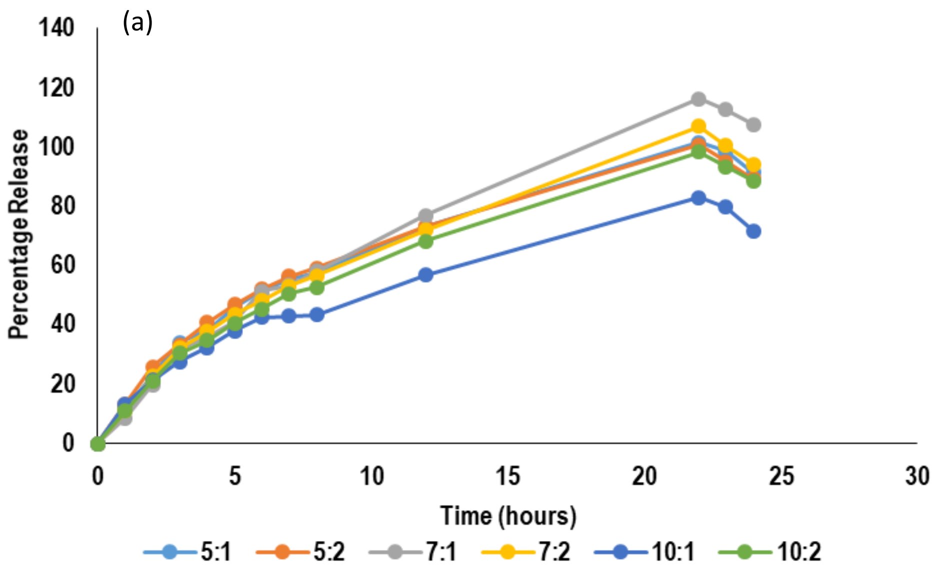
(a)
Percentage Release
Time (hours)
5:1
5:2
7:1
7:2
10:1
10:2

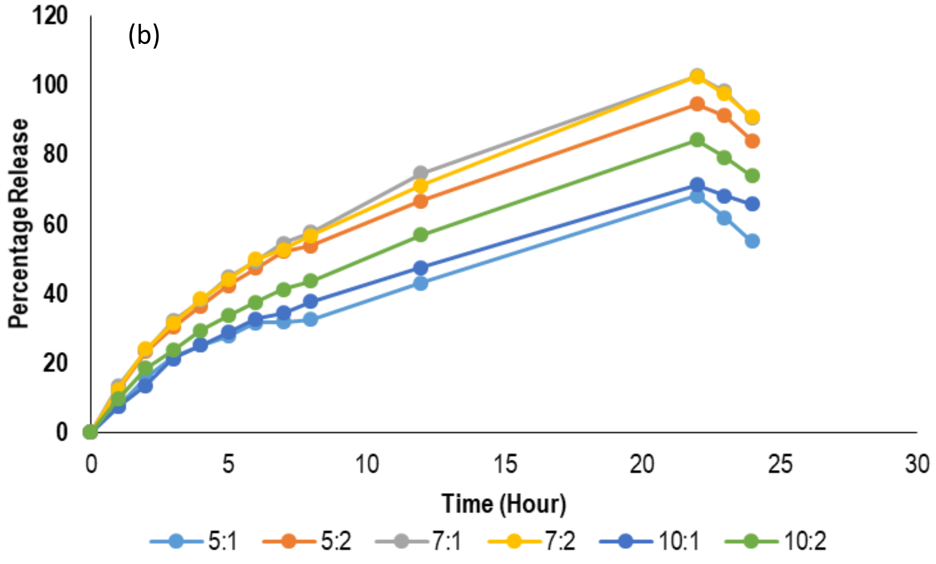
(b)
Percentage Release
Time (Hour)
5:1
5:2
7:1
7:2
10:1
10:2

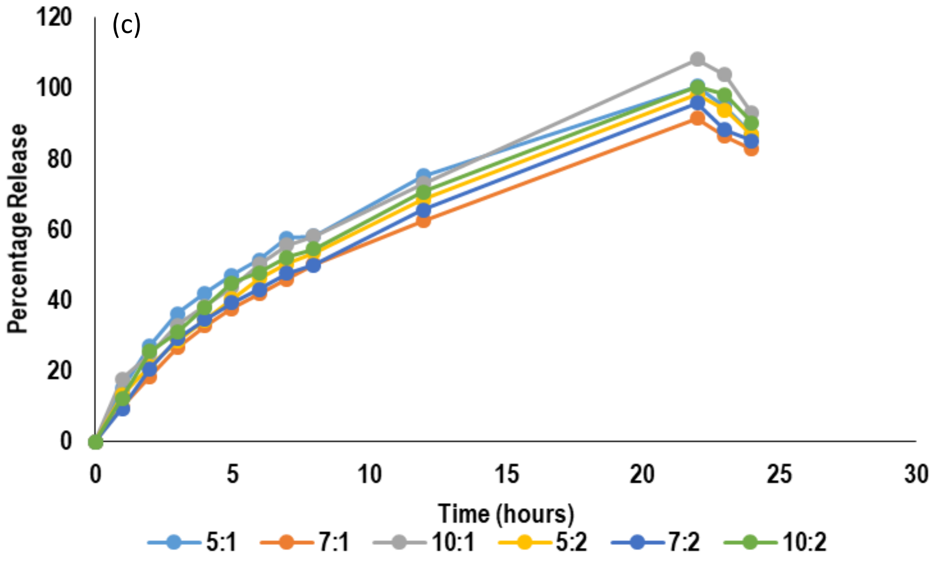

Figure S9-S11. Release study of lidocaine nanoemulsion formulated with (a) beeswax, (b) coconut oil and (c) oleic acid
